# Identifying the Potential Causes, Consequences, and Prevention of Communicable Diseases (Including COVID-19)

**DOI:** 10.1155/2020/8894006

**Published:** 2020-11-02

**Authors:** Muhammad Khalid Anser, Talat Islam, Muhammad Azhar Khan, Khalid Zaman, Abdelmohsen A. Nassani, Sameh E. Askar, Muhammad Moinuddin Qazi Abro, Ahmad Kabbani

**Affiliations:** ^1^School of Public Administration, Xi'an University of Architecture and Technology, Xi'an, China; ^2^Institute of Business Administration, University of the Punjab, Quaid-i-Azam Campus, Lahore, Pakistan; ^3^Department of Economics, University of Haripur, Khyber Pakhtunkhwa, Haripur, Pakistan; ^4^Department of Management, College of Business Administration, King Saud University, P.O. Box 71115, Riyadh 11587, Saudi Arabia; ^5^Department of Statistics and Operations Research, College of Science, King Saud University, P.O. Box 11451, Riyadh 11587, Saudi Arabia; ^6^Department of Management, Aleppo University, Aleppo, Syria

## Abstract

Communicable and noncommunicable diseases cause millions of deaths every year, increased billions of healthcare expenditures, and consequently increase trillions of economic losses at a global scale. This study more focused on the prevalence of communicable diseases, including COVID-19 that is an emerging pandemic, which affects the global economy. The objective of the study is to examine the impact of population density, lack of sanitation facilities, chemical concentration, fossil fuel combustions, poverty incidence, and healthcare expenditures on communicable diseases including COVID-19. The study covered a large panel of heterogenous countries to assess the relationships between the stated factors by using the robust least square regression, Granger causality test, and innovation accounting matrix. The study used a time series data from 2010 to 2019 for assessing the determinants of communicable diseases, while it is further extended with the current data of 2019-2020 for the COVID-19 pandemic. The results of the study show that high population density, lack of primary handwashing facilities, chemicals used in manufacturing value-added fossil fuel combustion, and poverty headcount substantially increase communicable diseases. In contrast, population diffusion, low carbon concentration in air, renewable fuels, and healthcare expenditures decrease infectious diseases in a panel of 78 countries. The causal inferences found the bidirectional relationship between communicable diseases and primary handwashing facility, and carbon emissions and poverty headcount, whereas the unidirectional relationship is running from lack of sanitation to infectious diseases, economic growth to carbon emissions, and communicable diseases to fossil fuel combustion across countries. Communicable diseases increase healthcare expenditures and decrease the country's economic growth which is a vital concern of the global economy to confront the outbreak of novel coronavirus through increasing the healthcare budget in national bills and stabilize financial activities at a worldwide scale.

## 1. Introduction

Communicable disease (also known as transmissible or infectious disease) is not a new word for the world as its roots can be traced back to the end of the First World War in 1918 when Spanish flu infected more than one-fourth of the world's population which resulted in 30-100 million causalities [1]. Indeed, this flu was a disaster for the entire world in the twentieth century. Since then, over a century, no epidemic has shocked the world by its causality in such a short period. It remained a fortune for the world that scientists successfully developed an embankment against both known and unknown transmissible diseases [2]. Innumerable informal and formal networks of organizations serving various people, having different objectives, accountability, modalities, and resources, across the globe participate in developing such systems. However, some of the new range of communicable disease threats such as COVID-19, Ebola, influenza, severe acute respiratory syndrome (SARS), Middle-East respiratory syndrome (MERS), the looming spectre of rising antimicrobial resistance (AMR), dengue, and Zika have challenged global health organization to provide a better health system to the world. Conjointly, these non- and unknown infectious diseases not only jeopardize human health but also endanger numerous economic and social prosperity. This is because of the lack of a single high-level entity that may have an eye on potential threats (caused by biological, accidental, or naturally occurring attacks, etc.), as well as on the network organization tasks with their vindication, investigation, and deterrence. Given that, this study is an attempt to identify causes, consequences, and prevention of infectious diseases (called outbreak, epidemic, or pandemic). Bloom and Cadarette [2] argued that “A sharp increase in prevalence in a relatively limited area or population is referred as outbreak, a sharp increase covering a larger area or population referred as epidemic, and an epidemic covering multiple countries is referred as pandemic” (p.2).

According to the United Nations [3] statistics, a significant disparity in longevity is observed between developed and developing countries. For instance, the life expectancy from birth decreased from 11 to 8 years for the developing countries (between 2000-2015), though remained persistent for high-income groups, whereas, in developed countries, life expectancy from birth raised from 65 to 69 years (for men) and 69 to 73 years (for women) between 2000 and 2015. Altogether, a 24 years increase in global life expectancy has been examined since 1950 [2]. This is because of a decline in infectious disease mortality because of improved hygiene, sanitation, vaccination, nutrition, availability of clean water, income, growth, health systems, medical practices, and antibiotics. Though humans claim that they managed to abate infectious diseases, these still are a threat to the world. The world is still fighting with old pathogens (such as plague and HIV) and new pathogens (like COVID-19). Similarly, some other diseases like malaria and tuberculosis (TB) are prevalent in many areas, whereas influenza and coronavirus (COVID-19) fluctuate in intensity and pervasiveness, inflicting mayhem in developed and developing countries. There still exist several unknown pathogens, which are the greatest challenge regarding responding and anticipating various epidemics. The World Health Organization [4] urged countries to work on the epidemic potential disease which was revised in 2018; however, in 2020, a new pandemic COVID-19 has shocked the world by hitting almost 202 countries within a short span of three months [5].

Still, there are many diseases for which no biomedical is available except precautionary measures; thus, WHO has suggested urgent researchers to make policies to escape out from the infectious diseases—for example, SARS, MERS, COVID-19, and Zika. Influential diseases (human-initiated or naturally occurring outbreaks or epidemics) are associated with many social and economic risks. First, such conditions cost a lot to the health system of any country regarding medical treatment. More specifically, an outbreak stresses the health system by limiting to deal with routine issues, whereas epidemics force infected individuals and their caretakers (e.g., physicians, scientists, and engineers) to stay at home, hence disrupting their productivity. Second, fear of infection may result in the closing of public services, transportation, commercial establishments, enterprises, and schools to create social distance, which are socially valuable economic activities [6]. A relatively contained outbreak can restrict a country's export, international tourism, and foreign direct investment. Influenza roughly costs US$ 500 billion worldwide, and dengue (in particular) costs US$ 56 million to Malaysia in 2010, where several Southeast Asian countries banned international tourism [7]. Furthermore, long-running epidemics (e.g., malaria and HIV) directly damage foreign direct investment [8]. Third, mortality as a result of outbreaks and epidemics directly affect a country's gross domestic product (GDP). Each year, the flu kills almost 646,000 people across the globe [9], which could result in a loss of 5% of the global GDP [10]. For example, the Ebola outbreak occurs in a decline in 8% GDP of West Africa between 2013 and 2014 [3], where 28616 morbidities and 11310 mortalities were identified; however, it is difficult to understand how much impact COVID-19 would have on the world's GDP especially on the Chinese economy.

This study is a need of the current time that calls for urgent attention to confront the challenges of a communicable disease, including COVID-19. This study is an initiative to contribute to the unprecedented time to stand with the healthcare professionals and government bodies to work together to fight against COVID-19. The study helps in the given literature through some most straightforward assumptions and evaluates the potential causes, consequences, and prevention of communicable diseases, including COVID-19.

The objective of the study is as follows:

- To determine the impact of population density, lack of primary handwashing facilities, chemicals used, carbon emissions, fossil fuel combustion, and poverty headcount on the prevalence of communicable diseases across countries
- To analyze economic losses by an increasing cause of death by infectious diseases, including COVID-19
- To investigate the incidence of communicable diseases including COVID-19, carbon emissions, fossil fuel combustion, and economic growth on healthcare expenditures
- To evaluate the role of population diffusion, handwashing availability, fewer chemicals used, low carbon emissions, renewable fuels, and healthcare expenditures in lessening communicable diseases across countries

The study used econometric panel techniques to assess the possible causes, consequences, and prevention of communicable diseases, including COVID-19 across countries.

## 2. Materials and Methods

### 2.1. Study Design

A large panel of 78 countries has been selected in this study for estimation. The robust least square regression, Granger causality test, impulse response function, and variance decomposition analysis are employed to empirically estimate the data for conclusive findings.

### 2.2. Data Sources

The data of the variables are taken from different sources, including the World Bank [11], POVCAL net, country demographic and healthcare surveys, and UNICEF [12]. The data of different variables are derived from the actual variable's data set due to the nonavailability of their given data for a more extended time. For instance, the COVID-19 data set for affected people and deaths are just available latest for a few months, while we assumed few values extracted from the given data set of communicable diseases in terms of observing the level of intensity of possible coronavirus that causes death across countries.

### 2.3. Description of Variables

COVID-19 has assumed some inherent values of coronavirus ranging from the lowest value 1 to the highest value 4. The small magnitude value (i.e., 1) of COVID-19 is assigned next to communicable diseases that fall in a range of 1% to 20% cause of death by contagious diseases, while value 2 is assigned to 20.1% to 40% of the total deaths caused by communicable diseases. The value 3 is allocated close to infectious diseases that fall in the range of 40.1% to 60% of the total deaths, and finally, the highest value 4 is assigned to communicable diseases that show a more significant number of deaths falling in the range of 60.1% to 80% caused by infectious diseases. The numerical illustration is shown in equation (1) as follows:

(1) $$\begin{array}{c} \text{COVID}‐19=\left\{\begin{array}{c} \text{Highest value }4\left\{\begin{array}{c} 60.1\%\text{cause of death by communicable diseases}\left(\%\text{of total death}\right)\\ 80\%\text{cause of death by infectious diseases}\left(\%\text{of total death}\right) \end{array}\right.\\ \text{Lowest value }1\left\{\begin{array}{c} 1\%\text{cause of death by communicable diseases}\left(\%\text{of total death}\right)\\ 20\%\text{cause of death by communicable diseases}\left(\%\text{of total death}\right) \end{array}\right. \end{array}\right.. \end{array}$$

The rationale to assign the lowest to the highest value of COVID-19 is that we assumed that the countries that have less death caused by communicable diseases have a less chance to spread coronavirus while the countries that have a greater magnitude in terms of more significant death caused by infectious diseases have a higher chance to spread coronavirus. This supposition would be helpful to fill the COVID-19 data against the selected panel of 78 countries. The data of communicable disease cause death in the percentage of total deaths is taken from the World Bank [11] data set and denoted by COMD.

Furthermore, the study used a data set of population density and split into three different data set to perceive the “population diffusion” data that is not available in any other form at the aggregate level. The population diffusion was assessed at three arbitrary levels, i.e., 0.25, 0.50, and 0.75. The PDIF_0.25_ shows an absolute population density of the rural areas, while PDIF_0.50_ and PDIF_0.75_ shows a random population density at medium and high urban density areas, respectively. We extract this data set from a given population density assigned its given weight at the stated three different levels. The numerical illustration shown in equation (2) is given below, i.e.,

(2) $$\begin{array}{c} \text{Population diffusion}\left(\text{PDIF}\right)=\left\{\begin{array}{c} {\text{PDIF}}_{0.25}=\text{Population density}\times 0.25\;\left(\text{rural density}\right),\\ {\text{PDIF}}_{0.50}=\text{Population density}\times 0.50\;\left(\text{medium density}\right),\\ {\text{PDIF}}_{0.75}=\text{Population density}\times 0.75\;\left(\text{suggested optimal size of urban population density}\right). \end{array}\right. \end{array}$$

The rationale to divide the population density into the given three population diffusion levels is that communicable diseases including COVID-19 are spread mainly in those countries and places where the population is highly compact; thus, “social distancing” suggested a way to escape out of coronavirus. The study gets benefited from this idea and splits the population density into three population diffusion levels, and it would like to see how much the population can diffuse to flee from COVID-19. The data of population density (people per square km of land area) is borrowed from the World Bank [11] data set and denoted by PDEN.

The data of “basic handwashing facilities including soap and water” as a percentage of the population is given in the World Bank [11] database, which is denoted here with the symbol of HSWF. The study gets benefited with this data and found the percentage of the population that has a lack of necessary handwashing facilities, which is denoted here by a symbol of LBHWF. The numerical expression is shown in equation (3), i.e.,

(3) $$\begin{array}{c} \text{LBHWF}=100\%-\text{HSWF}\%. \end{array}$$

The rationale to use this data is to assess how much the population has a lack of necessary handwashing facilities. The data set of HSWF of a few countries was not available at the World Bank database; thus, the data of urban sanitation as the percentage of the population is used for the given data, which is taken from the UNICEF [12] database.

The study used the data set of the “chemicals used” in manufacturing services as a percentage of its value added from the World Bank [11] database, which is denoted here with a symbol of CHM. The study takes out the data of the “low chemical used in the manufacturing sector” from CHM, which is indicated by LCUSE. The LCUSE is estimated by the semiportion of the chemical used in the manufacturing process, i.e.,

(4) $$\begin{array}{c} \text{LCUSE}=\frac{1}{2}\left(\text{CHM}\right). \end{array}$$

The rationale of LCUSE is to assess the need for chemicals used in the manufacturing sector that have to be filled with some alternative fuels relative to a high percentage of chemicals used in manufacturing value added, which turned into low cost and goods chemical purity.

The study is taken from “carbon emissions” data as metric tons per capita (denoted by CO_2_). In contrast, the data is taken out of the “low carbon contamination” in the air (denoted by LCC) from total carbon emissions with some alpha value that can be obtained from the carbon emission coefficient value in the regression apparatus. The alpha value is somewhere found between +1 and -1 to get the low carbon contamination in the atmosphere. The data is borrowed from the World Bank [11] database. The numerical illustration of LCC is as follows:

(5) $$\begin{array}{c} \text{LCC}={\text{CO}}_{2}\times \alpha^{+1}\left\{∴-1>\alpha \leq 1\right.. \end{array}$$

The data of poverty headcount ratio in the percentage of the population (denoted by POV), GDP per capita in constant 2010 US$ (denoted by GDPPC), energy use as kilograms of oil equivalent per capita (denoted by EUSE), and total health expenditure per capita in US$ (denoted by HEXP) are taken from the POVCAL NET (poverty data) and World Bank [11] database. The poverty data is used at national level estimates, while in some countries where the information is not available at the World Bank, then it has borrowed from the POVCAL NET database at US$1.90 poverty line.

Finally, the study used fossil fuel combustion data as a percentage of total energy (denoted by FFUEL). The renewable fuel (denoted by RF) data extracted from FFUEL is as follows:

(6) $$\begin{array}{c} \text{RF}=100\%-\text{FFUEL}\%. \end{array}$$

The data is taken from the World Bank [11] database.

### 2.4. List of Sample Countries

Table 1 shows a list of countries that used a study for ready reference.

### 2.5. Research Framework

**Figure 1:**
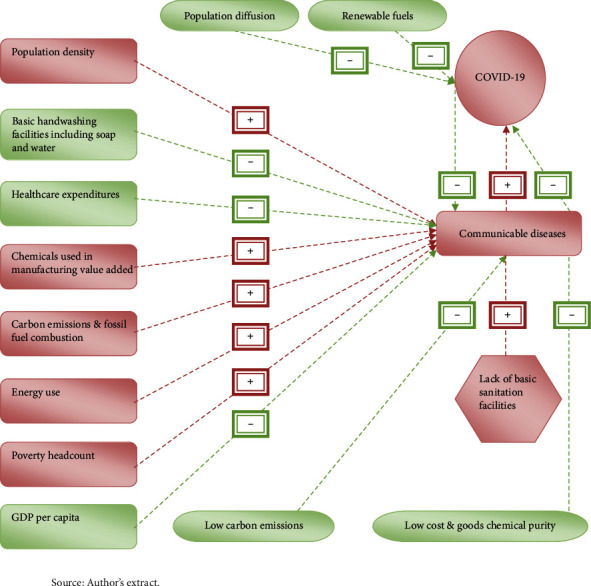
Research framework of the study source the authors extract.

The study is to get benefited from the earlier studies of Saleem et al. [13], Qureshi et al. [14], Aldakhil et al. [15], Batool et al. [16], and Majeed and Ozturk [17]. These studies mainly provoked the need for sustainable healthcare policies to mitigate adverse environmental externalities across the globe.  Figure 1 shows the research framework of the study for ready reference.

Figure 1 shows that population density, lack of sanitation facilities, chemicals used, fossil fuel combustion, energy demand, and poverty headcount will likely increase communicable diseases, including COVID-19. In contrast, necessary handwashing facilities, healthcare expenditures, GDP per capita, population diffusion, low carbon emissions, low cost and good chemical purity, and energy use will likely to decrease communicable diseases including COVID-19.

### 2.6. Research Hypotheses

Based on the suggested research framework, the study proposed the following research hypotheses, i.e.,

*H1*. It is the likelihood that lack of necessary handwashing facilities and population density both increase communicable diseases, including COVID-19.

*H2*. Carbon emissions, fossil fuel combustion, and poverty headcount are expected to increase communicable diseases including COVID-19.

*H3.* Population diffusion, energy use, low carbon emissions, low cost and good chemical purity, healthcare expenditures, and economic growth will likely decrease communicable diseases, including COVID-19.

### 2.7. Econometric Framework

The given hypotheses need an empirical survey to evaluate these stated factors to get more insights about the causes, consequences, and prevention of communicable diseases across countries. The study utilized a robust panel least square regression to minimize possible outliers from the criterion variable and its regressand. The MM estimator is used for addressing potential outliers in the given models. The previous studies mainly used this technique to get robust inferences, including Ozturk [18], Naz et al. [19], Rasli et al. [20], Zaman [21], and Aldakhil et al. [22]. Furthermore, the study used a causality test performed by the Wald test to evaluate the cause-effect relationship between the variables. Finally, the study used impulse response function (IRF) and variance decomposition analysis (VDA) to observe the error shocks on predictor variables to the response variable. The 10 years variance error shocks are estimated to find the forecast relationship between the variables across countries. The EViews version 9.0 software is used for empirical estimation.

## 3. Results and Discussion

Table 2 shows the descriptive statistics of the variables. The average percentage of deaths caused by communicable diseases is 12.921 with a maximum value of 71.5%, and the minimum value is 1.2%. The population density, necessary handwashing facilities, carbon emissions, and chemicals used in manufacturing value added are about 144.774 people per square kilometer of land area, 72.509%, 5.557 metric tons per capita, and 9.634%, respectively. The energy demand, fossil fuel combustion, and GDP per capita have a maximum value of 18178.144 kg of oil equivalent per capita, 99.977%, and US$110742.3 while an average value is 2583.704 kg, 72.927%, and US$17237.62, respectively. The mean value of health expenditures per capita and poverty headcount is about US$1463.884 and 15.084% of the population, respectively. The COVID-19 value shows that the average value is 1.277, which shows that deaths caused by communicable diseases majorly falls in the range of 1% to 20%.

Table 3 shows the robust least square regression estimates and found that population density increases communicable diseases and decreases the country's economic growth with an elasticity estimate of 0.043%, *p* < 0.050 and − 0.067%, *o* < 0.050, respectively. The result implies that the highly dense population areas where the people reside in a very compact place have a more incidence of spreading communicable diseases. Kolata [23] argued that infectious diseases, including coronavirus, spread rapidly mainly in the high-density area, the constant movement of international tourists, and keeping reliance on using public transports. These three stated factors need to be reformed to prevent it from transmitting diseases. McDonough [24] further endorses the need to cope with infectious diseases as it is likely to spread person-to-person more quickly in high population urban dense areas. Neiderud [25] highlighted the lack of adequate metropolitan city planning to avoid global healthcare challenges, including the epidemiology of infectious diseases. Communicable diseases are most likely to transmit in urban dense areas through close contact easily. The surveillance, public awareness programs, and some control programs may be helpful to decrease the burden of infectious diseases.

Adequate city planning would be helpful to reduce the risk of communicable diseases, as the regression estimates show that the estimates of PDIF_0.75_ minimize the impact of communicable diseases in urban areas. The other projection of population diffusion, i.e., PDIF_0.25_, shows that the rural population also may be affected through commutable diseases by close social networking. Thus, the judgment that rural areas could not or less affected by communicable diseases due to a healthy immune system required more careful assessment of the rural regions, while there is a need to share control programs to get less affected by the infectious diseases with the rural population. Carey [26] concluded that rural communities are at low risk of potential infectious diseases; however, there is a need to follow some precautionary measures as suggested by the state to avoid close contact with other people. The urban environment is compact mainly, and due to overcrowding, the transmission of diseases more likely to quickly spread in popular cities.

The other regression estimates show that the emergence of infectious diseases including COVID-19 increases healthcare expenditures, which negatively effect on country's economic growth. The elasticity estimates show that if one percent increases in communicable diseases, then healthcare expenditure increases by 0.433%, *p* < 0.000, while the country's economic growth decreases by -0.477% *p* < 0.000. Coronavirus could increase billions of healthcare expenditures in the national healthcare bills that are further expected to escalate insurance healthcare premiums by more than 40% as today in the next years [27]. Thus, it could negatively affect the country's economic activities. Aamir [28] argued that the coronavirus damages the country's healthcare system, as a fragile healthcare system would ultimately increase economic costs across the globe. Mendoza and Linderman [29] identified different critical challenges of shortage of medical care supplies including COVID-19 testing swabs, surgical equipment, protective masks, surgical gowns, and hand antiseptics; all these items increase healthcare import bills that affect the country's economic activities.

The regression estimates show that the “lack of basic hand-washing facilities” increases the risk of communicable diseases, which negatively affect the country's economic growth. The elasticity estimates of the stated relationship show that if there is a 1% increase in the lack of sanitation facilities, it will become cumbersome in the economy as 0.103%, *p* < 0.000 increase in the form of causes of death by communicable diseases and -0.243%, *p* < 0.000 decreases the country's economic growth. The availability of handwashing facilities would reduce the incidence of coronavirus up to 12.6% out of 100%, i.e., considering the great variability of handwashing facilities (min. 0.916; max. 99); the countries are not equally benefited by this protective factor. Healthcare inequalities and wide-spread poverty are considered the main cause to the increase in the infected coronavirus cases across the globe [30]. UNICEF [31] shared a substantial knowledge about how to minimize the risk of COVID-19 to the parents and common people and argued that the infectious diseases mostly spread by touching the face, coughing, sneezing, and respiratory droplets of an infected person; thus, there is a need to do some precautionary measures to minimize the risk of communicable diseases including COVID-19, i.e., frequent handwashing with water and soap, wearing a mask, social distancing, and seeking medical care if the flu, headache, and respiratory problems are still persistent. These precautions are vital to reduce the incidence of coronavirus across the globe.

The other direct measures that likely increase the incidence of communicable diseases are estimated in this study, i.e., chemicals used in manufacturing, fossil fuel combustion, and poverty incidence. The regression estimates show that these stated factors consider the main determinants of the increase in communicable diseases across countries. The elasticity estimates show that if there is a 1% increase in chemicals used, fossil fuel combustion, and poverty incidence, then the infectious diseases increase by 0.137%, *p* < 0.000; 0.311%, *p* < 0.000; and 0.110%, *p* < 0.000, respectively. The investment in renewable fuel projects and the increase in healthcare expenditures would reduce the burden of communicable diseases on a global scale. The earlier literature largely confirmed these factors as jeopardy of an increase in infectious diseases across countries; for instance, Movalli et al. [32] emphasized the need to perform chemical legislation as it is widely documented that human health and the natural environment both are primarily exposed with various chemicals used in production. Thus, the lack of different chemical regulations is favorable to the environment and human health. Perera [33] concluded that children's health is threatened mainly with fossil fuel combustion that impacts on their cognitive and behavioral impairment, which further leads to respiratory infections and other infectious diseases. There is a need for technology and health interventions to mitigate the adverse concerns of fossil fuel combustion across countries. Huda et al. [34] concluded that low socioeconomic settings, overcrowding, lack of sanitation and water facilities, and poverty lead to an increase in multiple communicable diseases, including hepatitis, dengue, and tuberculosis. The results argued that community-based involvement in reducing healthcare concerns and the role of government to reduce healthcare inequalities would be the vital factor for improving healthcare awareness across countries.

Finally, the regression estimates are mainly significant in terms of analyzing the consequences of communicable diseases, which is trace out by introducing COVID-19 variables in the study, and found that COVID-19 decreases healthcare expenditures when economic growth is increasing, while on the other hand, COVID-19 increases healthcare expenditures that lead to decrease the countries' economic growth in different regression apparatus. Brinza [35] stated that the coronavirus damages the country's economic activities through a substantial increase in healthcare expenditures, out-of-pocket health care expenditures, and expected future insurance premiums. All these factors could more worsen if the coronavirus is still exacerbated. Herper [36] highlighted the global healthcare issues that are visible after the new coronavirus, including the overcrowding of billions of the global world population, mixed human behaviors, and insufficient global healthcare mechanisms; all these factors lead to inviting infectious diseases in the fragile healthcare environment.  Table 4 shows the VAR Granger causality estimates for ready reference.

The results confirmed the feedback hypothesis between the communicable diseases and primary handwashing facilities with soap and water, and carbon emissions and poverty headcount. The result implies that the risk of infectious diseases primarily is minimized with frequent handwashing while lacking sanitation cumbersome pressure on healthcare expenditures that could increase death by communicable diseases. The bidirectional causality relationship between poverty incidence and carbon emissions implies that both factors move in the same direction. The high carbon emissions negatively influenced the poorer health, while due to the lack of knowledge, income, and vulnerability lead to degrading the environment. Thus, both factors need urgent attention to recorrect it by sustainable healthcare policies across countries. The unidirectional causal relationships confirmed that the causality relationship is running from (i) lack of sanitation to communicable diseases, (ii) adequate handwashing facility to economic growth, (iii) carbon emissions to population density and healthcare expenditures, (iv) healthcare expenditures to chemical use, (v) low carbon emissions to population density, (vi) energy demand to population density, (vii) GDPPC to carbon emissions, (viii) communicable diseases to fossil fuel, and (ix) COVID-19 to fossil fuel. The causal inferences would be helpful to support long-term sustainable healthcare policies on a global scale.  Table 5 shows the IRF estimates and found that communicable diseases will likely increase with high population density and lack of necessary handwashing facilities. At the same time, the remaining factors would be powerless to increase infectious diseases due to sustainable reforms over a time horizon.

The IRF estimates further show that COVID-19 will increase health care expenditures for the next two coming years since 2020-2021, i.e., the costs will decrease with the reducing number of cases. Communicable diseases and carbon emissions though increase healthcare expenditures for the next 10 years' time period. Furthermore, the population density and energy demand will likely decrease the country's economic growth in an upcoming 10 years' time period, while the chemicals used in the manufacturing process and healthcare expenditures would increase economic growth over a time horizon. The impact of COVID-19 for the next coming 5 years from 2020 to 2025 will vanish, and it will primarily damage the country's economic growth. Finally, the result shows that adequate sanitation facilities and low chemicals used would likely decrease communicable diseases, which further join hands with low carbon concentration for at least the next coming five years. At the same time, population density and healthcare expenditures will substantially increase communicable diseases over a time horizon.  Table 6 shows VDA estimates for ready reference.

The result shows that the lack of primary handwashing facility will exert a greater magnitude in terms of influenced communicable disease with a variance error shock of 6.142%, while the least affected will be carbon emissions with a variance error shock of 0.018%. The estimate for GDP per capita shows that carbon emissions will be influenced mainly by healthcare expenditures, followed by the country's economic growth, communicable diseases, and COVID-19, while the least changed will be fossil fuel combustion with a magnitude of 0.073%. Further, it shows that population density, chemical used, and energy demand will be of the greater magnitude to influence a country's economic growth. Finally, the estimates show that the primary handwashing facilities have a greater magnitude to influence on communicable diseases, while the least influence will be low carbon emissions over a time horizon.

The following are the possible limitations of the study that would possibly be added in future researches, i.e., firstly, the study assigned some numerical values for assessing COVID-19 variables while it can further be assessed through COVID-19-infected cases, registered death cases, recovered cases, and case fatality ratios. Secondly, the study used a large panel of countries in the estimation, while an individual country-based assessment may give more analytical wisdom to understand the COVID-19 pandemic. Finally, the study used a few panel econometric techniques to evaluate the coefficient values for robust inferences, which can be expanded with some more simulation techniques that may be helpful to devise long-term policy implications on a global scale.

## 4. Conclusions

Healthcare concerns have now become global phenomena, which connected countries to combat the epidemic of communicable and noncommunicable diseases through mutual coordination, cooperation, and technology transfers. The outbreak of coronavirus has disrupted the whole world, which closes the countries altogether to form unified healthcare policies to defeat this unwanted epidemic disease. The results show that high population density leads to an increase of communicable diseases through close contact among the resident members due to social binding, which led to a decrease in the country's economic growth. The spread of communicable diseases puts a burden on healthcare infrastructure in the form of high urgency of surgical instruments, including protective masks, hand sanitizers, testing swabs, and surgical gowns. The use of chemicals, fossil fuel combustion, and carbon pressure further exert pressure on different microorganisms that spread in the air which could infect people. The marginalized population is largely affected by communicable diseases, including COVID-19, as they are directly exposed to infectious diseases due to illiteracy and poverty. The causal inferences confirmed the two-way relationship between communicable diseases and sanitation, and carbon emissions and poverty, whereas the one-way cause relationship confirmed the income-led emissions and infectious diseases caused by fossil fuel across countries. The forecast relationship suggested that population density and lack of sanitation will likely to increase communicable diseases for the next 10 years' time period. Carbon emissions and communicable diseases will largely escalate healthcare expenditures over a time horizon. Furthermore, a lack of sanitation facilities will exert a greater magnitude in terms of influencing communicable diseases, which will increase healthcare expenditures across countries. It is sensible to be vigilant and keep yourself against danger rather than be casual.

## Figures and Tables

**Table 1:** List of countries.

Total countries: 78 Time period: 2010-2019	“Albania, Algeria, Armenia, Australia, Austria Azerbaijan, Bahrain, Belarus, Belgium, Bolivia, Bosnia and Herzegovina, Brazil, Bulgaria, Canada, Chile, China, Colombia, Costa Rica, Croatia, Cyprus, Ecuador, Egypt, Estonia, Ethiopia, Finland. France, Georgia, Greece, Hungary, Iceland, India, Indonesia, Iran, Ireland, Italy, Japan, Jordan, Kazakhstan, Kenya, Korea, Kyrgyz Republic, Latvia, Lithuania, Luxembourg, Malaysia, Malta, Mauritius, Mexico, Mongolia, Morocco, Namibia, New Zealand, Panama, Peru, Philippines, Poland, Portugal, Romania, Russia, Senegal, Serbia, Slovak Republic, Slovenia, South Africa, Spain, Sri Lanka, Sweden, Tanzania, Thailand, Tunisia, Turkey, Ukraine, UK, USA, Uruguay, Vietnam, Yemen, and Zimbabwe.”

**Table 2:** Descriptive statistics.

Statistics	COMD (%)	PDEN (people per square kilometer of land area)	HSWF (%)	CO_2_ (metric tons per capita)	CHM (%)	EUSE (kg of oil equivalent per capita)	FFUEL (%)	GDPPC (US$)	HEXP (US$)	POV (%)	COVID-19 (1-4 values)
Mean	12.921	144.774	72.509	5.557	9.634	2583.704	72.927	17237.62	1463.884	15.084	1.277
Maximum	71.500	2017.274	99	23.811	47.931	18178.14	99.977	110742.3	9871.742	72.300	4
Minimum	1.200	1.750	0.916	0.075	0.545	244.437	4.573	341.554	15.126	0.010	1
Std. dev.	15.263	262.737	22.076	4.589	6.579	2592.949	22.514	19233.49	1916.420	14.897	0.691
Skewness	2.220	4.563	-1.173	1.549	2.356	3.083	-1.267	1.926	1.813	1.221	2.581
Kurtosis	7.262	26.197	3.730	5.566	12.083	16.413	4.189	7.648	5.888	4.706	8.857

**Table 3:** Robust least-square regression estimates.

Variables	ln(COMD)	ln(GDPPC)	ln(HEXP)	ln(COMD)
Constant	1.588^∗^	2.423^∗^	-4.271^∗^	61.118^∗^
ln(PDEN)	0.043^a^^∗∗^	-0.067^c^^∗∗^		-
ln(PDIF_0.25_)	-	-		52.753^∗^
PDIF_0.50_	-	-		3.91E-05
ln(PDIF_0.75_)_*t*−1_	-	-		-52.755^∗^
ln(COMD)		-0.447^c^^∗^	0.433^g^^∗^	-
ln(LBHWF)	0.103^∗^	-0.243^c^^∗^		-
ln(HSWF)	-	-		-0.126^∗∗^
ln(CHM)	0.137^∗^	0.021		-
ln(LCUSE)	-	-		0.174^∗^
ln(CO_2_)	-0.726^∗^	-	1.195^f^^∗^	-
ln(LCC)	-	-		-0.050^d^^∗^
ln(FFUEL)	0.311^∗^	-	0.003	-
ln(POV)	0.110^b^^∗^	-		-
ln(EUSE)	-	0.223^∗^		-
ln(RF)	-	-		-0.068^d^^∗^
ln(HEXP)	-	0.730^∗^		-0.183^∗^
ln(GDPPC)	-	-	1.200^∗^	-
COVID-19	-	-0.122^∗^	-0.065^e^^∗∗^	-
Statistical tests				
*R*^2^	0.873^h^	0.803	0.813	0.784^e^
Rw^2^	0.981^h^	0.970	0.979	0.891^e^
Rn^2^	574.220^∗^	17854.99^∗^	13552.19	1179.98^∗^

Note: ^∗^ and ^∗∗^ indicate 1% and 5% significance level. ^a^The estimates found after removing CO_2_ and FFUEL in a given regression. ^b^The estimates found after removing CO_2_ in a given regression. ^c^The estimates found after removing EUSE and HEXP in a given regression. ^d^The estimates found after removing the PDIF variables in a given regression. ^e^The estimates found after removing CO_2_ and FFUEL. ^f^The estimates found after removing GDPPC, ^g^The estimates found after removing GDPPC. ^h^The aggregated value of *R*^2^.

**Table 4:** VAR Granger causality by the Wald test.

Bidirectional relationship	Unidirectional relationship	
∑COMD↔∑HSWF	∑LBHWF→∑COMD	∑HEXP→∑CHM
∑CO_2_↔POV	∑HSWF→∑GDPPC	∑HEXP→∑LCUSE
	∑CO_2_→∑PDEN	∑LCC→∑PDEN
	∑CO_2_→∑HEXP	∑EUSE→∑PDEN
	∑GDPPC→∑CO_2_	∑COVID→∑FFUEL
		∑COMD→∑FFUEL

Note: → shows the one-way causal relationship and ⟷ shows the bidirectional causal relationship.

**(a):** 

Response of COMD							
Period	COMD	PDEN	LBHWF	CHM	CO_2_	FFUEL	POV
1	1.009728	0	0	0	0	0	0
2	0.971082	0.012298	0.262409	-0.010020	0.032316	-0.028466	0.006108
3	0.931074	0.020977	0.258162	-0.013299	0.013421	-0.043322	-0.015149
4	0.907859	0.031815	0.248270	-0.015049	0.012690	-0.043405	-0.022032
5	0.885095	0.043558	0.242994	-0.017865	0.008563	-0.043342	-0.029479
6	0.862543	0.056683	0.237839	-0.020499	0.005273	-0.043461	-0.036453
7	0.840431	0.071265	0.232703	-0.023157	0.002082	-0.043655	-0.043159
8	0.818757	0.087476	0.227635	-0.025810	-0.000831	-0.043912	-0.049528
9	0.797506	0.105488	0.222634	-0.028471	-0.003486	-0.044244	-0.055578
10	0.776667	0.125498	0.217694	-0.031147	-0.005855	-0.044661	-0.061307

**(b):** 

Response of HEXP						
Period	HEXP	COVID-19	COMD	CO_2_	FFUEL	GDPPC
1	161.1844	0	0	0	0	0
2	156.2567	0.804499	3.462596	9.846654	1.803069	-1.196444
3	156.5138	-0.553185	4.240749	9.390322	0.463866	-1.894477
4	157.1644	-1.988518	5.035252	10.90567	-0.746911	-3.659306
5	157.7119	-3.350708	5.813208	12.03504	-1.983548	-5.657835
6	158.2858	-4.684526	6.560719	13.23044	-3.222137	-7.828792
7	158.8597	-5.983756	7.286742	14.42421	-4.468027	-10.09466
8	159.4376	-7.251429	7.993291	15.63375	-5.721767	-12.43274
9	160.0184	-8.488917	8.682373	16.85836	-6.984202	-14.83246
10	160.6019	-9.697829	9.355435	18.09925	-8.255852	-17.29032

**(c):** 

Response of GDPPC								
Period	GDPPC	COMD	LBHWF	CHM	PDEN	EUSE	HEXP	COVID-19
1	643.9326	0	0	0	0	0	0	0
2	895.0244	11.21296	-1.185111	-33.81734	-21.69921	-20.93595	16.04855	-9.879289
3	994.0067	14.74853	2.930395	-7.158432	-52.19020	-23.94878	18.14983	-10.71273
4	1037.941	14.62711	4.767937	35.55866	-88.29733	-30.55231	18.42102	-8.380977
5	1061.072	13.36505	5.505336	84.65466	-128.5741	-36.44630	18.36574	-4.781985
6	1076.869	11.89280	5.850025	135.8865	-172.8341	-42.59936	17.78438	-0.751515
7	1090.320	10.48397	6.092191	187.7078	-221.3182	-49.01128	16.85133	3.392802
8	1103.337	9.249303	6.330860	239.5871	-274.4744	-55.75366	15.56168	7.506505
9	1116.667	8.227222	6.616580	291.3639	-332.8696	-62.88187	13.90029	11.51120
10	1130.619	7.430071	6.980254	343.0227	-397.1622	-70.45130	11.83706	15.35104

**(d):** 

Response of COMD							
Period	COMD	HSWF	LCUSE	LCE	RF	HEXP	PDEN
1	1.010723	0	0	0	0	0	0
2	0.975074	-0.266702	-0.008598	-0.033431	0.029162	-0.002528	0.007956
3	0.934093	-0.262988	-0.011574	-0.013449	0.043283	0.001650	0.014013
4	0.907904	-0.252393	-0.012583	-0.009900	0.043274	0.006939	0.021189
5	0.883101	-0.245768	-0.014695	-0.005294	0.042752	0.011426	0.029323
6	0.858720	-0.239557	-0.016792	-0.001226	0.042499	0.015690	0.038535
7	0.834943	-0.233442	-0.018965	0.002503	0.042370	0.019781	0.048930
8	0.811768	-0.227462	-0.021204	0.005821	0.042355	0.023691	0.060635
9	0.789178	-0.221616	-0.023515	0.008721	0.042466	0.027415	0.073792
10	0.767154	-0.215894	-0.025902	0.011182	0.042712	0.030947	0.088559

**(a):** 

VDA of COMD								
Period	SE.	COMD	PDEN	LBHWF	CHM	CO_2_	FFUEL	POV
1	1.009728	100	0	0	0	0	0	0
2	1.426027	96.50846	0.007437	3.386126	0.004937	0.051354	0.039847	0.001835
3	1.723370	95.26757	0.019909	4.562504	0.009336	0.041227	0.090476	0.008983
4	1.964591	94.66370	0.041545	5.107879	0.013051	0.035897	0.118435	0.019489
5	2.169584	94.26304	0.074373	5.442651	0.017482	0.030991	0.137020	0.034442
6	2.348302	93.95251	0.121748	5.671538	0.022543	0.026958	0.151211	0.053496
7	2.506867	93.68230	0.187649	5.838422	0.028314	0.023724	0.163013	0.076582
8	2.649389	93.42457	0.277017	5.965389	0.034840	0.021250	0.173417	0.103512
9	2.778821	93.16084	0.395919	6.064516	0.042168	0.019474	0.182990	0.134096
10	2.897405	92.87658	0.551784	6.142775	0.050343	0.018321	0.192078	0.168116

**(b):** 

VDA of HEXP							
Period	S.E.	HEXP	COVID-19	COMD	CO_2_	FFUEL	GDPPC
1	161.1844	100	0	0	0	0	0
2	224.7462	99.77376	0.001281	0.023737	0.191952	0.006436	0.002834
3	274.0761	99.70107	0.001269	0.039902	0.246460	0.004614	0.006684
4	316.1970	99.61319	0.004908	0.055338	0.304128	0.004025	0.018415
5	353.6656	99.51027	0.012900	0.071251	0.358901	0.006363	0.040312
6	387.8730	99.38566	0.025311	0.087848	0.414738	0.012191	0.074254
7	419.6436	99.23732	0.041956	0.105201	0.472464	0.021751	0.121302
8	449.5212	99.06404	0.062586	0.123301	0.532702	0.035158	0.182208
9	477.8866	98.86516	0.086931	0.142107	0.595787	0.052467	0.257553
10	505.0196	98.64025	0.114716	0.161564	0.661929	0.073705	0.347839

**(c):** 

VDA of GDPPC									
Period	S.E.	GDPPC	COMD	LBHWF	CHM	PDEN	EUSE	HEXP	COVID-19
1	643.9326	100	0	0	0	0	0	0	0
2	1103.745	99.79191	0.010321	0.000115	0.093873	0.038650	0.035979	0.021141	0.008012
3	1486.715	99.70348	0.015529	0.000452	0.054058	0.144534	0.045779	0.026556	0.009608
4	1816.118	99.47868	0.016894	0.000992	0.074562	0.333237	0.058979	0.028084	0.008568
5	2109.444	99.03838	0.016536	0.001417	0.216319	0.618515	0.073569	0.028397	0.006865
6	2379.085	98.34924	0.015499	0.001718	0.496301	1.014021	0.089899	0.027913	0.005407
7	2633.610	97.39768	0.014233	0.001937	0.913004	1.533698	0.107995	0.026873	0.004578
8	2879.154	96.17873	0.012941	0.002104	1.456381	2.192067	0.127859	0.025406	0.004510
9	3120.346	94.69164	0.011713	0.002241	2.111835	3.004286	0.149468	0.023615	0.005201
10	3360.908	92.93805	0.010585	0.002363	2.862014	3.986046	0.172777	0.021595	0.006569

**(d):** 

VDA of COMD								
Period	S.E.	COMD	HSWF	LCUSE	LCE	RF	HEXP	PDEN
1	1.010723	100	0	0	0	0	0	0
2	1.430235	96.41951	3.477252	0.003614	0.054637	0.041575	0.000312	0.003095
3	1.729061	95.15691	4.692598	0.006954	0.043433	0.091111	0.000305	0.008685
4	1.969840	94.55915	5.257222	0.009438	0.035990	0.118460	0.001476	0.018263
5	2.173385	94.18698	5.597347	0.012325	0.030158	0.136004	0.003976	0.033206
6	2.349939	93.91924	5.827089	0.015649	0.025824	0.149042	0.007859	0.055294
7	2.505750	93.70524	5.992873	0.019491	0.022812	0.159675	0.013144	0.086762
8	2.644996	93.51789	6.118043	0.023919	0.020957	0.168948	0.019819	0.130420
9	2.770659	93.34035	6.215451	0.029002	0.020090	0.177461	0.027852	0.189792
10	2.884979	93.16046	6.292631	0.034810	0.020032	0.185595	0.037196	0.269276

## Data Availability

The data is freely available at “worldometer” and “world development indicators”.
